# Surface structure, model and mechanism of an insect integument adapted to be damaged easily

**DOI:** 10.1186/1477-3155-2-10

**Published:** 2004-10-01

**Authors:** Jean-Luc Boevé, Véronique Ducarme, Tanguy Mertens, Philippe Bouillard, Sergio Angeli

**Affiliations:** 1 Department of Entomology, IRSNB-KBIN, Royal Belgian Institute of Natural Sciences, Rue Vautier 29, B-1000 Bruxelles, Belgium; 2 Present address: Unité d'écologie et de biogéographie, Croix du Sud, 4–5, B-1348 Louvain-la-Neuve, Belgium; 3 Unité de modélisation des structures et des matériaux, CP 194/5, Université Libre de Bruxelles, Avenue Roosevelt 50, B-1050 Bruxelles, Belgium; 4 Institut für Zoologie, Stephanstrasse 24, Justus-Liebig-Universität Giessen, D-35390 Giessen, Germany; 5 Present address: Institut für Forstzoologie und Waldschutz, Georg-August Universität Göttingen, Büsgenweg 3, D-37077 Göttingen, Germany

## Abstract

**Background:**

Several sawfly larvae of the Tenthredinidae (Hymenoptera) are called easy bleeders because their whole body integument, except the head capsule, disrupts very easily at a given spot, under a slight mechanical stress at this spot. The exuding haemolymph droplet acts as a feeding deterrent towards invertebrate predators. The present study aimed to describe the cuticle surface, to consider it from a mechanistic point of view, and to discuss potential consequences of the integument surface in the predator-prey relationships.

**Results:**

The integument surface of sawfly larvae was investigated by light microscopy (LM) and scanning electron microscopy (SEM) which revealed that the cuticle of easy bleeders was densely covered by what we call "spider-like" microstructures. Such microstructures were not detected in non-easy bleeders. A model by finite elements of the cuticle layer was developed to get an insight into the potential function of the microstructures during easy bleeding. Cuticle parameters (i.e., size of the microstructures and thickness of the epi-versus procuticle) were measured on integument sections and used in the model. A shear force applied on the modelled cuticle surface led to higher stress values when microstructures were present, as compared to a plan surface. Furthermore, by measuring the diameter of a water droplet deposited on sawfly larvae, the integument of several sawfly species was determined as hydrophobic (e.g., more than Teflon^®^), which was related to the sawfly larvae's ability to bleed easily.

**Conclusion:**

Easy bleeders show spider-like microstructures on their cuticle surface. It is suggested that these microstructures may facilitate integument disruption as well as render the integument hydrophobic. This latter property would allow the exuding haemolymph to be maintained as a droplet at the integument surface.

## Background

The integument of insects is very often involved in defence strategies towards predators and pathogenic agents [1,2]. Generally it constitutes the first contact point in the interaction between an insect and such natural enemies. It often offers an efficient protection as a physical barrier due to its hardness, for instance, in adult beetles. At the opposite extreme, a low mechanical strength of the integument can be implicated in insect defence strategies as well. One example of this is the phenomenon of reflex bleeding that is known in several insect orders. The integument presents a few localized weak points which can disrupt when the insect under disturbance will increase its internal hydraulic pressure, provoking the release of a droplet of distasteful haemolymph [e.g., [3]]. The phenomenon of easy bleeding is another type of adaptation used in defence, by where the whole body integument, except the head capsule, can disrupt easily at a given spot when this spot is subjected to mechanical stress [see definition in [4]]. The phenomenon occurs in the larvae of some species belonging to sawflies (Hymenoptera, Symphyta, Tenthredinidae). Species that show easy bleeding notably belong to genera such as *Aneugmenus*, *Athalia*, *Monophadnus*, *Phymatocera *and *Rhadinoceraea*. Recently, the mechanical strength of dissected pieces of larval integument was measured in a calibrated manner. The force needed to damage the integument can vary in more than one order of magnitude from one species to another [4]. Easy bleeding differs from reflex bleeding in that, first, almost the whole body integument is potentially involved in the phenomenon, and second, an external force is necessary to exhibit the phenomenon [4]. As soon as the integument of an easy bleeder is damaged, a haemolymph droplet exudes and can remain as such during several minutes.

An ecological implication of easy bleeding is that the emission of a haemolymph droplet will deter an attacking predator from killing and feeding on an easy bleeder. Indeed, the haemolymph is feeding deterrent towards foraging ants and wasps [4-8]. Birds are other important predators of sawfly larvae [9], but to which easy bleeding seems less clearly effective [10]. Thus the ecological function of easy bleeding is demonstrated as a chemically mediated defence strategy directed especially towards foraging invertebrate predators.

However, integument disruption remains puzzling from a morphological and mechanistic point of view. The present study is based on a comparative analysis of the larval integument surface in several sawfly species, which comprise easy bleeders as well as non-easy bleeders. We wanted to describe the geometry and to approach the mechanical properties of the integument surface, and to consider proximate, ecological implications.

## Results

### Microstructures covering the cuticle surface

**Figure 1:**
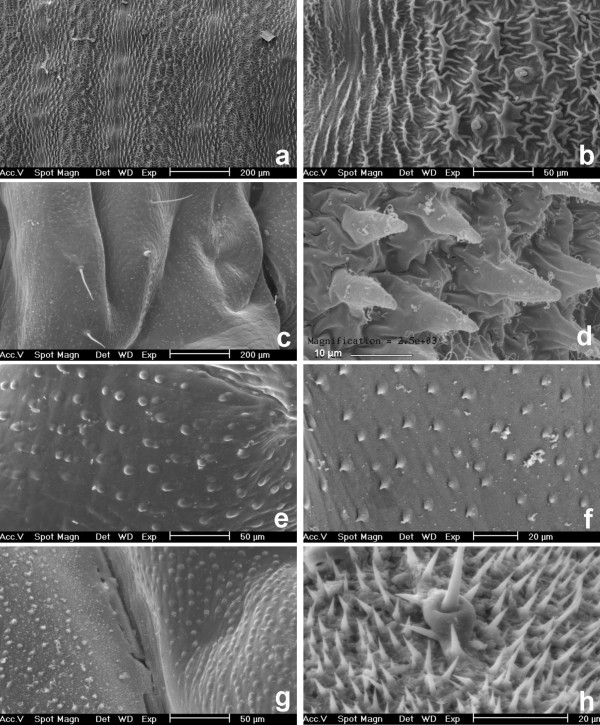
**Cuticle surfaces of sawfly larvae by SEM. **Easy bleeders are *A. rosae *(a, b) and *M. monticola *(d). Non-easy bleeders are *C. septentrionalis *(c), *H. australis *(e), *N. miliaris *(f), *P. parvula *(g) and *G. hercyniae *(h). The dorso-lateral part of the abdomens is shown. Detailed view showing spider-like microstructures (b). Views showing blister-like swellings (c, e to g) or setae (h).

**Figure 2:**
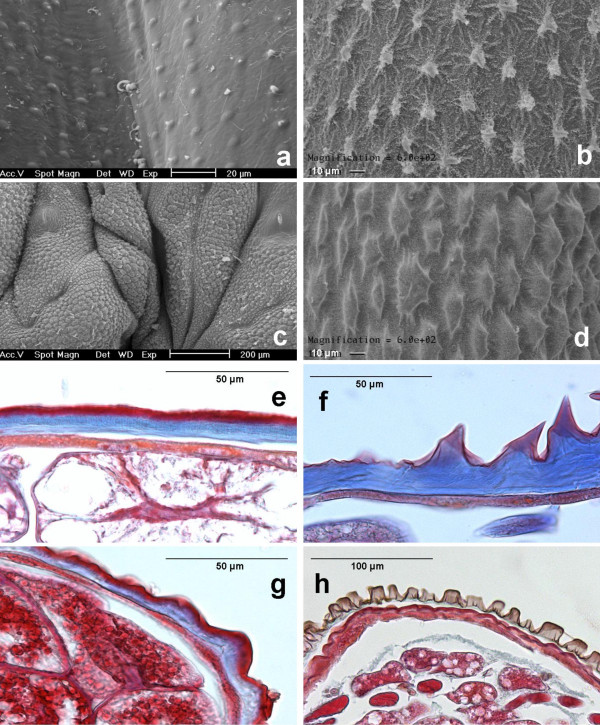
**Cuticle surfaces of sawfly larvae by SEM and related integument sections by LM. **Non-easy bleeder is *S. multifasciata *(a, e). Easy bleeders are *P. aterrima *(b, f), *A. padi *(c, g), *R. nodicornis *(d) and *R. bensoni *(h). Views by SEM (a to d) show blister-like swellings (a) or spider-like microstructures (b to d). Views by LM (e to h) showing that, above a cellular layer, the cuticle comprises a procuticle, in blue, whereas the epicuticle, in red (e, g), is not observed in some species (f, h).

The larvae of sawfly species observed by SEM showed above surface microstructures of their cuticle and which are described below. These microstructures were strikingly more complexly structured in easy bleeders than in non-easy bleeders (Fig. 1, 2) and this differing occurrence among sawfly species was significant (*P *= 0.0001, Fisher exact probability test, N = 24 species; Table 1).

**Table 1:** Easy bleeding, cuticle microstructures and hydrophobic property in sawfly larvae

**Species**	**Easy bleeding^1^**	**Microstructures^2^**	**Droplet^3^** **2 μl**	**Diameter^3 ^** **4 μl**
**TENTHREDINIDAE**				
**Allantiinae**				
*Athalia rosae *(L.)	EB	+	?	? or 2.1 ± 0.0
**Blennocampinae**				
*Eurhadinoceraea ventralis *(Panzer)	EB	-	·	·
*Monophadnus monticola *(Hartig)	EB	+	·	·
*Monophadnus spinolae *(Klug)	EB	-	·	·
*Phymatocera aterrima *(Klug)	EB	+	? or 1.5 ± 0.0	? or 2.0 ± 0.0
*Rhadinoceraea bensoni *Beneš	EB	+	·	·
*Rhadinoceraea micans *(Klug)	EB	+	?	?
*Rhadinoceraea nodicornis *Konow	EB	+	? or 1.6 ± 0.1	? or 2.0 ± 0.1
*Tomostethus nigritus *(Fabricius)	N-EB	-	·	·
**Nematinae**				
*Craesus alniastri *(Sharfenberg)	N-EB	-	1.6 ± 0.0	2.1 ± 0.0
*Craesus septentrionalis *(L.)	N-EB	-	1.7 ± 0.0	2.2 ± 0.1
*Hemichroa australis *(Serville)	N-EB*	-	1.6 ± 0.1	2.2 ± 0.1
*Hemichroa crocea *(Geoffr.)	N-EB	-	·	·
*Hoplocampa testudinea *(Klug)	N-EB	-	·	·
*Nematus melanocephalus *Hartig	·	-	·	·
*Nematus miliaris *(Panzer)	N-EB*	-	·	·
*Nematus pavidus *Serville	N-EB*	-	·	·
*Pristiphora laricis *(Hartig)	N-EB	-	·	·
*Pristiphora testacea *(Jurine)	N-EB	-	1.9 ± 0.0	2.6 ± 0.0
*Pseudodineura parvula *(Klug)	·	-	·	·
**Selandriinae**				
*Aneugmenus padi *(L.)	EB	+	1.7 ± 0.2	2.2 ± 0.2
*Strongylogaster mixta *(Klug)	N-EB	-	1.7 ± 0.1	2.1 ± 0.1
*Strongylogaster multifasciata *(Geoffr.)	N-EB	-	1.6 ± 0.1	2.1 ± 0.0
**Tenthredininae**				
*Tenthredo scrophulariae *L.	N-EB*	-	·	·
**ARGIDAE**				
*Arge *sp.	N-EB	-	·	·
**DIPRIONIDAE**				
*Gilpinia hercyniae *(Hartig)	N-EB	-	1.6 ± 0.0	2.0 ± 0.0

^1 ^Species was an easy bleeder (EB), or a non-easy bleeder (N-EB). Data from Boevé & Schaffner [4], except data from U Schaffner & JLB, unpublished results (*).

^2 ^Spider-like microstructures were present (+) or absent (-) by observations of the cuticle surface by SEM and/or of cuticle sections by LM.

^3 ^Cuticle was either too hydrophobic so that adherence of water droplet was impossible (?) or the diameter (mean ± SD, in mm) of a 2 and 4 μl droplet on the cuticle was measured. (·) Not tested.

In easy bleeders, the cuticle is covered with irregularly shaped wart-like microstructures (verrucose). Their density is approximately of 15 units per 0.01 mm^2^. They possess fine ridges (carinulate) in a radiated way (Fig. 1b, 2b,2d), hence the term "spider-like". The fine ridges (i.e., the "legs" of the "spider") more or less imbricate in between those from adjacent microstructures, and their width is approximately of 0.5 to 1.5 μm. The form of the microstructure is generally circular (diameter excluding ridges: 10 μm in *A. padi*), but can be elongated (length: 35 μm in *A. rosae*). The ridges can be reduced (e.g., in *A. padi*). The height of microstructures was measured on LM views and reaches 23 μm (in *P. aterrima*). For further measurements by LM, see Table 2.

**Table 2:** Model input and output with force applied on cuticle of non-easy bleeders (A) and easy bleeders (B)

**A**								
	**M1/1**	**M1/2**	**M1/10**	**Hc**	**Pl**	**Pt**	**Sm**	**Ts**

**W1**	110	110	110	110	110	110	110	110
**H1**	20	20	20	7	5	8	8	11
**H2**	10	10	10	6	5	3	5	5
**E1**	500	500	500	500	500	500	500	500
**E2**	500	1000	5000	5000	5000	5000	5000	5000

**F 1z**								
**Max**	0.206	0.170	1.001	2.910	4.520	2.651	2.906	2.201
**Min**	-0.835	-1.003	-1.604	-4.167	-6.240	-6.482	-4.633	-3.772
**F 1x**								
**Max**	1.553	1.614	1.794	2.545	2.956	3.337	2.714	2.569
**Min**	-0.363	-0.383	-0.447	-0.717	-0.860	-0.864	-0.764	-0.710

**B**								

	**M2**	**Ar**	**Mm**	**Pa**	**Rb**	**Rn**		

**W1**	110	70	33.5	60	50	60		
**H1**	15	8	8	15	10	15		
**H3**	15	8	14	23	10	10		
**D1**	28	23	9	20	15	20		
**D2**	20	11	1	2	10	10		
**S1**	20	5	6	8	8	8		
**P**	1	3.306	80	100	4	4		
**N μstr**	1	1	5	1	1	1		

**F 1z**								
**Max**	0.665	1.696	0.623	4.291	1.430	0.770		
**Min**	-0.793	-2.560	-0.972	-7.286	-1.412	-2.134		
**F 1x**								
**Max**	4.788	7.554	35.820	174.600	12.840	7.632		
**Min**	-1.476	-2.241	-1.782	-9.267	-3.805	-2.317		

Model of non-easy bleeders was based on parameter values measured on LM and SEM views from *H. crocea *and *N. pavidus *together (M1/1, M1/2, M1/10), and from *H. crocea *(Hc), *P. luridiventris *(Pl), *P. testacea *(Pt), *S. multifasciata *(Sm) and *T. scrophulariae *(Ts). Different relative values of Young's modulus for procuticle (E1) and epicuticle (E2) were used in M1/1, M1/2, and M1/10.

Model of easy bleeders was based on parameter values measured on LM and SEM views from *P. aterrima *and *R. micans *together (M2), and from *A. rosae *(Ar), *M. monticola *(Mm), *P. aterrima *(Pa), *R. bensoni *(Rb) and *R. nodicornis *(Rn). Parameter values, in μm, introduced in the model: width of the model sample (W1), height of procuticle layer (H1), height of epicuticle layer (H2), height of microstructure (H3), diameter at base of microstructure (D1), diameter at top of microstructure (D2), shortest distance between microstructures (S1). Number of microstructures set under pressure (N μstr). Pressure applied per microstructure (P).

Stress values, obtained with a normal force (F 1z) or shear force (F 1x), are given as extreme values in traction (Max) and compression (Min).

Compared to easy bleeders, the cuticle surface of non-easy bleeders was much smoother. It only shows blister-like swellings (pustulate) which have a diameter of 3–4 μm (e.g., in *T. nigritus*, *H. australis*, *Nematus*, Fig. 1e,1f), 6–7 μm (e.g., in *S. multifasciata*, Fig. 2a) up to 12 μm (in *H. testudinea*). In some genera such as *Nematus *and *Craesus*, each swelling shows a very small prickle (echinulate). Several swellings are sometimes aligned and then can be joined, several together, to form a low ridge of approximately 35 μm long (in *C. septentrionalis*).

Although *E. ventralis *and *M. spinolae *are easy bleeders, no spider-like microstructures were detected. Instead, small ridges with one or a few prickles, and small spines were observed, respectively. The larvae (alive) of these two species as well as *T. scrophulariae *are covered with a layer of waxy powder. Setae were observed instead of microstructures in the outgroup species *G. hercyniae *(Fig. 1h).

### Modelling the mechanical behaviour of the cuticle

**Figure 3:**
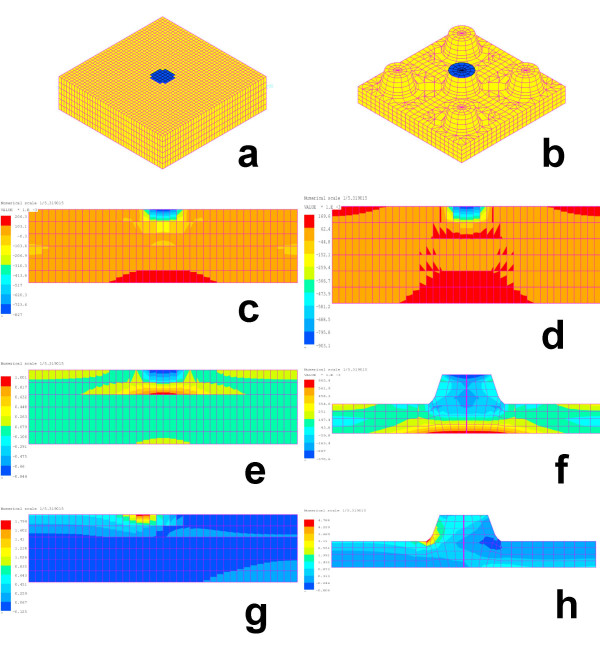
**Models of the cuticle of sawfly larvae. **Model representing a non-easy bleeder (a, c to e, g) and an easy bleeder (b, f, h). View in perspective showing five microstructures (b) and the location of the applied force (a, b). Maximal stress distribution in a section through the cuticle (c to h). The ratio of Young's modulus for the procuticle to the one of the epicuticle is assumed to be 1/1 (c), 1/2 (d) and 1/10 (e). The applied force is normal (c to f) or sheared (g, h). The maximal value corresponds to the maximal stress of the principal stress 1 and the minimal value to the minimal stress of the principal stress 2. Only the distribution of principal stress 1 is shown, while the maximal value is given in Table 2. Degrees of freedom = 120,553 (a, c to e, g), 40,701 (b, f, h).

The aim of modelling was to compare the repartition of stresses of two cuticle configurations, as found in non-easy bleeders (M1) versus easy bleeders (M2), when a same loading is applied (Fig. 3a,3b). The maximum stress value (in compression and traction) is an indicator of possible initiation of crack or damage (see Methods).

In Table 2, M1/1, M1/2 and M1/10 show the influence of the Young's modulus of the epicuticle, relative to the procuticle, on the distribution of stresses in a cuticle patch. When the Young's modulus of the epicuticle was increased, keeping the one of the procuticle constant, the stresses concentrated in the epicuticle and the maximal values increased (Fig. 3c to 3e). This occurred with both load cases (i.e., normal and shear force).

With a normal force, M1 and M2 resulted in stress values which were in the same range of magnitude (Table 2, Fig. 3c to 3f). Thus from this load case it cannot be deduced that one integument configuration will be damaged more easily than the other. In contrast, stress values obtained with a shear force were approximately three times higher in M2 than M1 (Table 2; Fig. 3g,3h). This suggests that an integument with microstructures is more constrained and will more easily reach the yield stress corresponding to the damage of the cuticle. This conclusion was corroborated by considering several single sawfly species. The maximal stress value in compression as well as in traction was always more extreme in five species of easy bleeders than in five species of non-easy bleeders (Table 2, see shear force). Note that for the easy bleeders *M. monticola *and *P. aterrima*, the apical part of the microstructure was extremely minute. All stresses were concentrated in the tip of the microstructure, which lead to non-physical deflections. The obtained values for these two species are probably irrelevant in a comparison with other species.

### Hydrophobic property of cuticle surfaces

It was difficult or even impossible to deposit a water droplet on the larval body of some sawflies (Table 1). When the pipette tip was brought close to the integument, almost within physical contact, the droplet was pushed aside against the tip border. By then retrieving the pipette, the droplet was again on its tip, not on the integument. This could happen for some of the individuals tested per species (Table 1).

In species where the integument was less hydrophobic, the diameter of the droplet on it ranged from 1.5 to 1.9 mm (2 μl droplet) and from 2.0 to 2.6 mm (4 μl). Considering this latter droplet size, a small diameter (2.0 mm) or an immeasurable diameter (see above) was associated with sawfly species which are easy bleeders, whereas a larger droplet diameter (> 2.0 mm) was associated with non-easy bleeders (*P *= 0.045, Fisher exact probability test, N = 12 species, Table 1). Thus easy bleeders possess a hydrophobic and non-easy bleeders a rather hydrophilic integument.

On inert surfaces the diameter of 2 and 4 μl droplets was constantly as follows: immeasurable (see above) and 2.2 mm on Teflon^®^, 1.8 and 2.3 mm on Parafilm^®^, 1.8 and 2.3 mm on polystyrene and 2.6 and 3.3 mm on glass, respectively. Thus even Teflon^®^, that is considered as highly hydrophobic, led to a 4 μl droplet diameter which was comparable to the one obtained on the (hydrophilic) integument of non-easy bleeders. The 2 μl droplet was apparently light enough to impede its adhesion on the Teflon^® ^surface, but not the larger droplet size tested.

## Discussion

Several sawfly larvae showed a characteristic cuticle surface with spider-like microstructures and this was associated with a low mechanical strength of their integument. For instance, these microstructures were present in the easy bleeder *Aneugmenus padi *and absent in the non-easy bleeders *Strongylogaster *spp. Both genera are closely related since they belong to the same subfamily and have the same host plant [11]. It is likely that the occurrence of microstructures cannot be interpreted simply in terms of a systematic arrangement of species and that they are related to the phenomenon itself of easy bleeding. The larval abdomen of several *Dolerus *(Tenthredinidae, Selandriinae) species presents "meshes of microsculpture not sharply defined", with a dimension ranging from 20 to 40 μm, and these microstructures are often fused [12]. They may constitute an intermediate state between those observed by us on easy bleeders and non-easy bleeders, but being more physically comparable to those of non-easy bleeders by the absence of spider-like microstructures. Particular microstructures are also observed at the cuticle surface of other arthropods than sawflies, such as in nymphs of bugs and ticks [13] and in adults of flies and dragonflies [14,15]. Their function is to allow by stretching an increase of body volume during feeding, to ally flexibility with mechanical stability during highly repeated movements, etc., but their possible role in promoting a mechanical damage of the integument was not envisaged so far [16].

The question arises to know whether in sawfly larvae able to bleed easily the microstructures are directly involved in integument disruption. We compared cuticle models of non-easy bleeders versus easy bleeders and applied a unit force on it. Compared to the real-life, the model was simplified by considering a linear elastic behaviour of the cuticle (i.e., the stresses are proportional to the strains – Hooke's law), because we do not know the exact physical properties of the cuticle. Nevertheless, a comparison of geometrical parameters from easy bleeders versus non-easy bleeders revealed that by applying a shear force the cuticle stresses both in compression and tension were higher in the presence of microstructures (Table 2). This suggests that microstructures may directly contribute in the damage of the integument. Yet, the breaking line of a damaged integument goes between the microstructures (SA, personal observation on the easy bleeder *P. aterrima*). This biological observation is in agreement with our model results. The regions subject to high stresses are not restricted to the zone of the microstructure, but extend deeper into the cuticle mass (Fig. 3h). From this trend we may extrapolate that if the shear force is enhanced, the microstructure will not break off from the rest of the cuticle, but the fracture line will start at the base of a microstructure and continue throughout the whole cuticle thickness. In other words, the integument will disrupt. This conclusion becomes even more relevant in the realistic situation where an attacking predator applies a more or less oblique force on the cuticle. Beside physical aspects, chemical ones also contribute in the mechanical properties of an integument [16-20]. One of these properties, visco-elasticity, is determined in the abdominal integument of the bug *Rhodnius *by the matrix protein(s) of the procuticle with a reinforcing effect of chitin microfibriles. Differing chitin and protein patterns are observed in the cuticle when easy bleeders are compared to non-easy bleeders (M. Spindler-Barth & SA, unpublished results). Ongoing research aims to investigate these physiological aspects as well as the healing process, and to link them with the phenomenon of easy bleeding.

The cuticle surface of easy bleeders was highly hydrophobic (Table 1) as compared to a well-known hydrophobic material such as Teflon^®^. There is a trend for the integument of easy bleeders (e.g., *P. aterrima*, *Rhadinoceraea *spp., *A. padi*) to appear as mat, in contrast to the brilliant aspect in non-easy bleeders (e.g., *Strongylogaster *spp., *Craesus *spp.) (JLB, personal observations). We believe that the hydrophobic property is ecologically relevant during predator-prey interactions. When a predator, typically an insect with biting-chewing mandibles [10], bites into the integument of a sawfly larva at a given spot, the best for the larva is to keep the deterrent haemolymph spatially concentrated at this spot. A counter-example is that some insects are known to have morphological devices of the integument surface or wetting agents included in their defensive secretion, which help the secretion to spread out [21,22]. But such secretions are typically volatile and the defence consists of keeping the aggressor at a distance. The morphological devices and wetting agents modulate the evaporation of the secretion and, thereby, the effectiveness of a defence that acts by olfactory cues. In the case of easy bleeding, deterrent compounds dissolved in the haemolymph need to contact the mouthparts of an aggressor, acting by gustatory cues. Moreover, easy bleeders should not spread out their hemolymph since they would lose this valuable liquid. Remaining as a droplet and in contact with the larval haemocel, the droplet can be sucked back by the larva into its body within a few minutes, providing that the larva is not more disturbed [4].

A parallel can be drawn between the integument surface of easy bleeders and the one of several plant leaves. The lotus leaf led recently to the so-called Lotus-effect^® ^[23]. Particular physico-chemical properties of the leaf allow a self-cleaning by rain. This effect relies on a micro-structured surface and a coating of waxy crystals. Both characteristics contribute in rendering the surface hydrophobic [23,24]. The optimal configuration and size of the structures is a coarse structure of 10 to 50 μm and a finer one of 0.2 to 5 μm [25]. This corresponds well to the case of the spider-like microstructures as found on the cuticle of easy bleeders. In the insect both these coarse and finer structures are provided by the spider-like structures (Results), whereas in the plant each scale of structures is due to microstructures and waxy crystals, respectively [26]. There are no waxy crystals on the body surface of easy bleeders. A fine layer of waxy powder covers only some species of easy bleeders as well as non-easy bleeders (see Results). Such a waxy powder consists mainly of hexacosan-1-ol in *Eriocampa ovata *[27], a non-easy bleeder [4] not studied in the present work. It is likely that in a majority of easy bleeders the hydrophobic property relies especially or solely on the geometry of the cuticle surface, by the occurrence of microstructures.

## Conclusions

We suppose at least two types of functions in the occurrence of spider-like microstructures, which we observed specifically on the body surface of easy bleeders. Firstly the damage provoked by a biting predator could be facilitated. Secondly the integument of easy bleeders could be rendered hydrophobic, which helps stop the emitted haemolymph droplet from spreading out.

## Methods

### Insects

All sawfly larvae (see Table 1) were collected in the field (Belgium, Germany, Switzerland), except *A. rosae *and *G. hercyniae *that came from indoor populations. The larvae were identified according to Lorenz & Kraus [11]. The full-grown larval stage was used.

### Observations by SEM and LM

Fixed larvae stored in ethanol were dried, coated with gold, and examined with a Philips XL-30 ESEM. Specimens were placed to observe the dorsal and lateral part of the abdomen. The terminology used in describing the cuticle surface refers to Harris [28].

Series of 7 μm thin cross sections were obtained from larvae by using classical histological techniques. They were deparaffinized in xylene and rehydrated in several decreasing ethanol to water solutions, then stained by the Azan trichrome method [29] and observed by LM.

### Model by finite elements

#### General mechanical assumptions

The general rigorous mechanical behaviour of the cuticle is complex. As a first attempt to understand the property of easy bleeding, it was assumed that a damage of the integument is due to excessive stress under static loading. Since the cuticle of a larva is also geometrically complex in three dimensions, no simplified laws, for instance, derived from the strength of material could be used. The analysis was therefore performed on solid configuration, discretized by a standard finite element method [30]. It was assumed that the stress-strain law is linear and isotropic (Hooke's law) and that the displacements and strains are small. The geometrical dimensions of the cuticle are very small, at the microscale. It is known that for such a configuration, the assumption of continuum may not be valid [31]. But, it is also known for standard materials such as metals that the strength is generally underestimated with continuum assumption. This is the reason why the analysis performed in this paper was purely qualitative and based on a comparison of the stress between the geometry encountered in easy bleeders and non-easy bleeders. Most of the results were interpreted on the principal stress: for 3D mechanical configurations, three directions always exist for which the stress (and strain for isotropic laws) is maximum or minimum. According to these directions, the shear stress is zero. It was then supposed that the maximum stress values (in traction or compression) cause the initiation of the cuticle damage.

#### Finite element modelling

The finite element analysis was performed using the general mechanical purpose software SAMCEF^® ^version 9.1. It was assumed that the integument is made of the repetition of reproducible patches in both x, y directions. Thus, only one patch has to be modelled by the appropriate boundary conditions representing this repetition (i.e., the displacements on each boundary are blocked in the direction normal to this boundary). The geometry was discretized with 3D solid linear finite elements (prisms or bricks). The patch used to model non-easy bleeders was composed of two layers, procuticle and epicuticle, of different properties in height and Young's modulus. It is supposed that generally the epicuticle of insects is pliant but not extensible, stronger in compression than tension, and that the epicuticle is less elastic than the procuticle [2]. The patch used for easy bleeders contained five microstructures and was homogenous. Indeed, generally no epicuticle is clearly detected in the cuticle of easy bleeders observed by LM (e.g., Fig. 2f), an exception being *A. padi *(Fig. 2g).

#### Loading

The loading was always divided into two load cases: a force perpendicular to the patch surface (normal force) and parallel to it (shear force).

In a real situation, the mandibles of an attacking predator, typically a small arthropod, apply the loading [10]. The diameter of a mandible's tip was measured on workers of the ant *Myrmica rubra *and reached 20 μm as the smallest value. In the model, the shape of the contact point made by the mandible was a disc (radius = 10 μm) on which the force was applied. This force was applied either on the upper centre of the epicuticle for non-easy bleeders (Fig. 3a) or on the top of the central microstructure when considering easy bleeders (Fig. 3b). As the radius of the upper part of the microstructure changed from one species to another, being generally lower than 10 μm, the applied surface force was adapted to obtain a same resultant force for each configuration.

The insect body contains a liquid, haemolymph. The patch, therefore, was modelled by applying a surface force equilibrated with the loading of the predator.

### Hydrophobic property

This property of the integument was estimated by a simple method that allowed the use of insects alive. The first step was to notice whether a 2 and 4 μl droplet of charcoal filtered water could adhere, gently depositing it with a 1–10 μl pipette on the thoracic or abdominal integument of a sawfly larva that was resting on a leaf of its host plant. If an adherence was possible, the diameter of the deposited droplet was measured under a stereomicroscope with micrometer. Six full-grown larvae were tested per species. As control the following substrates were tested in the same manner: Teflon^®^, Parafilm^®^, polystyrene and glass. On these biological and inert surfaces, the droplet reaction (i.e., adherence capability and droplet diameter) was considered to express the hydrophobic or hydrophilic property of the surface.

## Authors' contributions

JLB collected and identified the insects, performed the tests on the hydrophobic property, measured the cuticle parameters for the model on LM views, and wrote the manuscript, except the parts about this model in Results and Methods. VD obtained most SEM views. TM and PB performed the model by finite elements and wrote the two related parts in the manuscript. SA maintained indoor populations of two sawfly species and carried out and photographed the integument sections used in LM and, thereby, in the model.
